# Overcoming Airway Hurdles: A Case Report of Anesthetic Challenges in Meningomyelocele Complications

**DOI:** 10.7759/cureus.59192

**Published:** 2024-04-28

**Authors:** Pooja Thaware, Ankita, Anshu P Lakra, Jitendra Kushwaha, Mohd Yunus

**Affiliations:** 1 Trauma and Emergency Medicine, All India Institute of Medical Sciences, Bhopal, Bhopal, IND

**Keywords:** scoliosis, meningomyelocele, hydrocephalus, dexmedetomidine, conscious sedation, bronchoscopes

## Abstract

This case report delves into the anesthesia management in a 23-year-old male with complications of meningomyelocele, a catastrophic congenital neural tube defect. The patient, paraplegic since birth with severe scoliosis, presented with a femoral fracture, prompting the need for careful consideration of anesthesia strategies. The challenges included counseling the family on the risks and benefits of surgery under general anesthesia, selecting an appropriate anesthetic plan for an anticipated difficult airway, and addressing ventilation strategies for restrictive lung disease. To tackle the anticipated difficult airway, an awake pediatric fiberoptic bronchoscopy was performed in the recovery room, facilitating a conscious sedation approach. In the operating room, monitored anesthesia care with dexmedetomidine infusion was employed, complemented by careful positioning and padding in the lateral position. The awake fiberoptic checkscopy proved crucial in avoiding unnecessary general anesthesia. A patient-centered approach contributed to the successful execution of a complex anesthesia plan, ensuring optimal care for this unique patient population.

## Introduction

Meningomyelocele, or open spina bifida, is a catastrophic congenital neural tube defect associated with significant morbidity and various devastating complications [1]. This case report aims to explore detailed aspects of managing anesthesia in such cases, including challenges such as counselling family members, assessing risks and benefits, selecting a safe anesthetic plan for a challenging airway, and planning ventilation strategies for restrictive lung disease in the presence of severe scoliosis. The case discussed involves a 23-year-old male, paralyzed since birth, with a weight of 25 kg, length of 2 feet, and facing an expected difficult airway.

## Case presentation

A 23-year-old male, weighing 25 kg and length of 2 feet, is a paraplegic patient, with no independent walking or sitting abilities requiring assistance for mobility. Cognitive development is markedly delayed, functioning at a 5-year-old level, likely exacerbated by both neurological impairment and lack of formal education. Speech is characterized by stammering, akin to a 5-year-old's speech patterns. This patient presented to the emergency department after his mother noticed swelling over the right thigh. Following an X-ray, the patient was diagnosed with a fractured femur shaft, despite no reported history of trauma. He was scheduled for open reduction and internal fixation of the right femur.

The patient's medical history revealed that he was born with a lumbar meningomyelocele. At the age of four days, he underwent surgery for meningomyelocele repair, resulting in paraplegia with no motor power or sensory perception beyond the umbilicus. Additionally, there was a loss of bowel and bladder sensation. At 18 months, the patient underwent surgery for hydrocephalus, and a ventriculoperitoneal shunt was placed, indicating Arnold Chiari Type II malformation without brainstem dysfunction.

**Figure 1:**
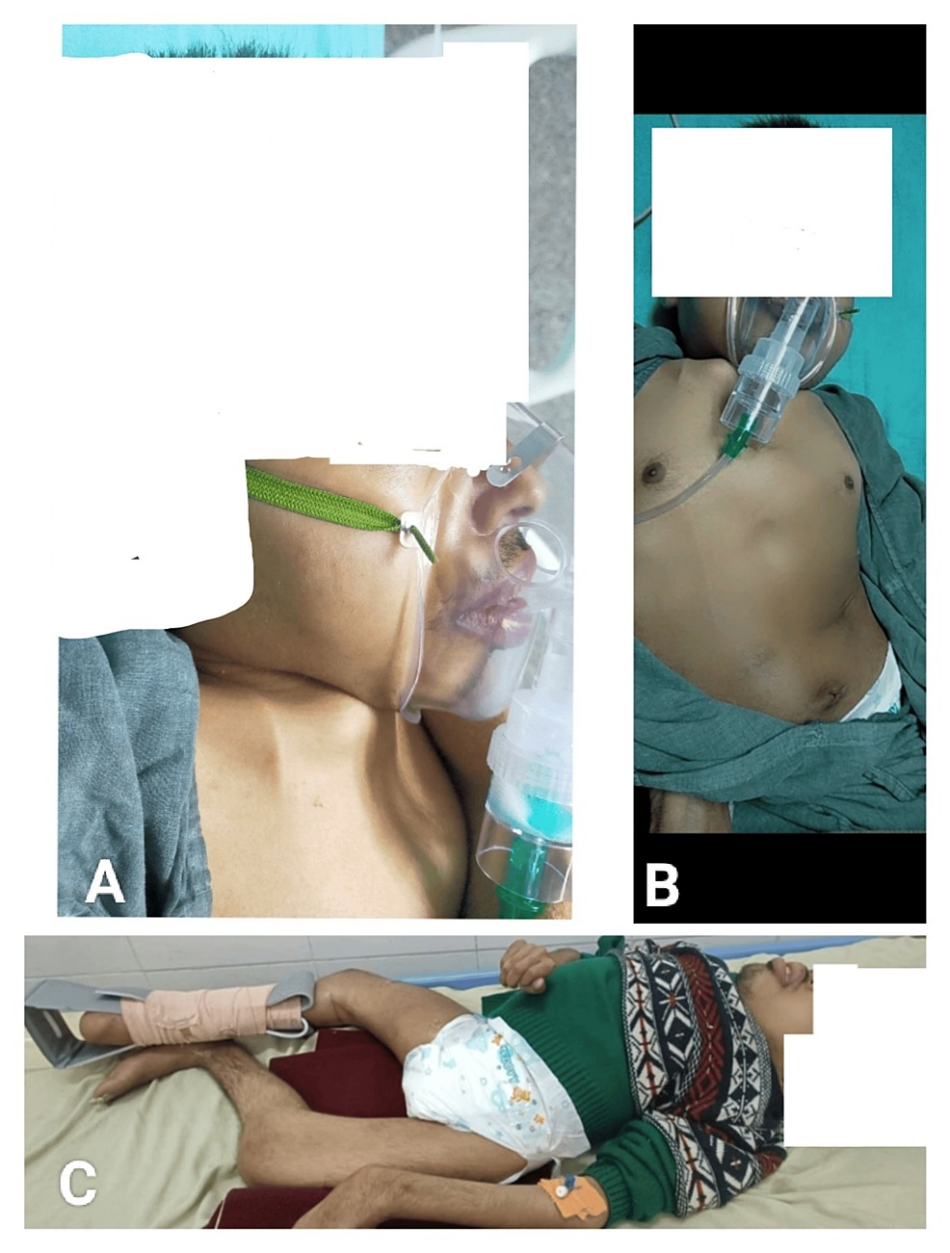
(A) Picture showing short fixed neck. (B) Picture showing short torso due to scoliosis. (C) Picture showing contractures

**Figure 2:**
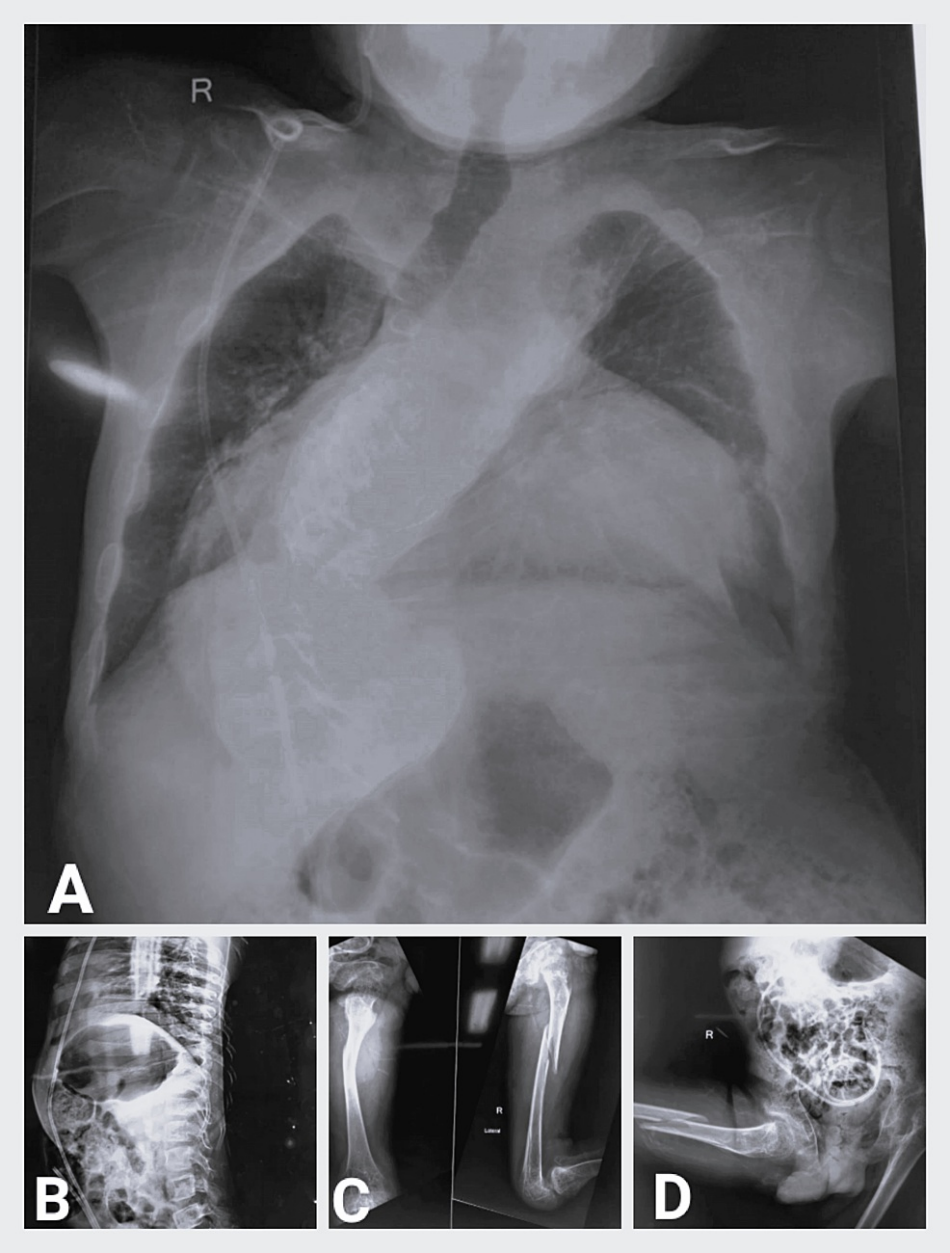
X-ray films of the chest, spine, femur, and pelvis (A) X-ray films of the chest showing scoliosis and deviated trachea toward the right. (B) X-ray thoracolumbar spine with abdomen showing ventriculoperitoneal shunt in situ. (C) X-ray anteroposterior and lateral view of the right femur showing femur shaft fracture. (D) X-ray pelvis with bilateral hip showing pelvic deformity along with ventriculoperitoneal shunt in situ and right femur shaft fracture

As the patient aged, severe scoliosis developed, occasionally causing difficulty in breathing. Two lower limb contracture release surgeries were performed under monitored anesthesia care. Contractures in the wrist and elbow joints also developed (Figure 1). Chest and spine X-ray and spine examination indicated severe scoliosis toward the right side, with a dimple and scar in the lumbar region (Figure 2).

Airway examination revealed an anticipated difficult airway with a short neck, chin touching the sternal notch, complete restriction of flexion and extension, a mouth opening of three fingers, upper lip bite test grade 3, Mallampati classification IV, and a buck teeth. The patient was able to talk with stammering, was cooperative, but exhibited anxiety and tremors. He expressed fear in the preoperative area before being taken for surgery.

A 2D echocardiography showed mildly dilated pulmonary arteries without any congenital heart disease or valve abnormality, with an ejection fraction of 60%. Abdominal ultrasound suggested calcification in the renal parenchyma. Laboratory investigations were within normal limits, except for a total bilirubin level of 1.2 mg/dL. Pulmonary function tests revealed severe restrictive lung disease. Arterial blood gas (ABG) analysis on room air suggested normal pO2 (103 mmHg) but decreased pCO2 (22 mmHg) due to tachypnea.

However, due to the contractures and the difficulty in achieving surgical exposure caused by paraplegia, the surgeons determined that the lateral position was necessary for the surgery. Although the patient had no pain sensation below the umbilicus and was paraplegic, theoretically allowing for the surgery to be performed under monitored anesthesia care, the severe anxiety and the lateral position requirements posed challenges, adding further complexity to an already difficult case.

Despite the calculated risks, the parents remained adamant about proceeding with the surgery. Their decision was respected, and in anticipation of potential complexities, an ICU bed was reserved for postoperative care.

The anesthesia plan was then decided to involve monitored anesthesia care and conscious sedation with a dexmedetomidine infusion, along with careful positioning and padding in the lateral position. A pediatric fiberoptic bronchoscope and videolaryngoscope were kept on standby on the difficult airway cart. The primary challenge identified was the potential difficulty in managing the airway if conscious sedation deepened, leading to airway collapse, or if the patient experienced a panic attack during surgery, necessitating a conversion to general anesthesia.

To address this concern, an awake oral fiberoptic checkscopy with a 4.5 mm pediatric fiberoptic bronchoscope was planned in the preoperative area to assess potential airway difficulty. In the recovery room, the patient received nebulization with 2 ml of 4% lignocaine under monitoring. Additionally, 10% lignocaine was sprayed on the posterior pharyngeal wall, and glycopyrrolate 0.1 mg was administered intravenously. The patient was thoroughly counselled about the diagnostic nature of the procedure and assured that it would not cause any pain. With the airway well anesthetized from lignocaine nebulization and spray, the patient underwent checkscopy without any difficulty. A small bite block was provided for the patient to hold between the teeth to prevent biting of the fibreoptic bronchoscope.

The pediatric fibreoptic bronchoscope passed through the oral cavity smoothly, and the vocal cords were visualized without resistance. The checkscopy took only 15 seconds, providing assurance for handling any airway complications during conscious sedation intraoperatively. The patient was then transferred to the operating room.

In the operating room, intravenous fluid (Ringer's Lactate) was started at 50 ml/hr through a 18 G intravenous cannula in the right arm. Standard monitors were attached, revealing a heart rate of 120/min due to anxiety, a respiratory rate of 30/min, and an oxygen saturation of 96% on air. Oxygen was administered via a face mask at 5 L/min. Since the patient maintained spontaneous ventilation, a sampling line was attached to the mask to obtain an end-tidal CO2 trace, ensuring the patient's breathing remained spontaneous and regular. A 0.5 mg intravenous injection of midazolam was given, and a dexmedetomidine infusion was initiated at 1 mcg/kg for 10 minutes. After sedation, vitals stabilized with a heart rate of 105/min, a respiratory rate of 25/min, and a saturation of 99% with 5 L/min oxygen flow. After 10 minutes, the dexmedetomidine infusion was reduced to 0.5 mcg/kg/h.

The patient was carefully padded and positioned in the lateral position to prevent additional injury. The dexmedetomidine infusion was continued and titrated between 0.2 to 0.7 mcg/kg/h. Intravenous 500 mg paracetamol was administered to alleviate any upper body pain. The surgery proceeded for two hours without any complications, and the patient was transferred to the postanesthesia care ICU for a 24-hour postoperative monitoring. The next day, the patient was transferred back to the ward.

## Discussion

Meningomyelocele is characterized by the failure of the neural tube to close in the lumbosacral region during embryonic development (fourth-week postconception), resulting in the herniation of the meninges and spinal cord through a vertebral defect [2-3].

Possible complications associated with meningomyelocele include learning disabilities, cognitive impairments, seizures, hydrocephalus, paralysis, loss of sensation below the lesion site, decreased mobility due to associated muscle weakness, neurogenic bladder, frequent urinary tract infections, bowel dysfunction, pressure ulcers due to sensory loss, and orthopedic problems such as scoliosis, contractures, and hip dislocation [4]. All these complications were exhibited in our patient except seizures, contributing to a poor quality of life. It is essential to note that femur fixation surgery is not likely to improve or change the patient's quality of life.

These complications presented a myriad of challenges, the first being the counselling of the attendants to make an informed decision regarding proceeding with anesthesia and surgery. Since the patient was already paraplegic, unable to sense pain, and incapable of walking, a conservative approach for femur fixation could have been considered to avoid the complications associated with anesthesia and surgery. The risks and benefits were explained to the relatives, encompassing perioperative management challenges such as a difficult airway leading to bronchospasm, laryngospasm complicating respiratory and cardiovascular hemodynamics, respiratory complications during difficult weaning if the patient undergoes surgery under general anesthesia, and an increased risk of postoperative infection, given the patient's bowel and bladder incontinence. Despite the risks outweighing the potential benefits, the affection of the parents toward their child did not allow this to happen, and they chose surgery.

Allaying anxiety and managing the difficult airway constituted the second challenge. Despite being 23 years old, the patient exhibited the maturity level of a child. He was counselled at each step and perioperatively cared for like a child. The use of awake flexible fiberoptic technology proved to be a boon for anesthesiologists in this case. Given the aim to avoid general anesthesia, deemed unnecessary for a femur fracture fixation due to the patient's lack of pain sensation below the umbilicus and the presence of restrictive lung disease resulting from severe scoliosis, awake flexible fiberoptic technology became an invaluable tool.

In our case, spinal anesthesia was never considered as an option, as the patient already had no sensation below the umbilicus. Even in other scenarios, the deformed spine due to scoliosis and the status post meningomyelocele surgery would have excluded spinal anesthesia as a viable choice. To implement conscious sedation and monitored anesthesia care as Plan A for this case, we needed to ensure the extent of airway difficulty, which we determined through preoperative pediatric fiberoptic bronchoscopy. Once completely sure, we proceeded with conscious sedation as Plan A. In the event of any complications, Plan B involved intraoperative supination of the patient and pediatric fiberoptic intubation. A videolaryngoscope was kept ready, and a Proseal laryngeal mask airway (LMA) number 3 was included on the difficult airway cart, considering the potential for high airway pressure due to severe scoliosis.

This case report also underscores the crucial role of early antenatal ultrasound examinations. While fetal surgery is an option, it comes with expenses and risks and is only offered at a few centers [5-6].

Approximately 75% of patients with spina bifida survive to early adulthood. An interprofessional, patient-centered, team-based approach to providing medical, educational, social, and developmental services can enhance these patients' quality of life by improving their overall health and functioning [7].

## Conclusions

This case underscores the delicate decision-making involved in managing meningomyelocele complications. The primary message emphasizes the nuanced balance between benefits and risks, necessitating informed discussions with families for optimal outcomes. The secondary message highlights the adaptability of awake fiberoptic technology in anesthesia, showcasing the importance of tailoring strategies to the unique needs of meningomyelocele patients. Overall, this case report contributes valuable insights, emphasizing the importance of personalized, multidisciplinary care for individuals with meningomyelocele.
